# Microfluidics for genome-wide studies involving next generation sequencing

**DOI:** 10.1063/1.4978426

**Published:** 2017-03-10

**Authors:** Sai Ma, Travis W. Murphy, Chang Lu

**Affiliations:** 1 Department of Biomedical Engineering and Mechanics, Virginia Tech, Blacksburg, Virginia 24061, USA; 2 Department of Chemical Engineering, Virginia Tech, Blacksburg, Virginia 24061, USA

## Abstract

Next-generation sequencing (NGS) has revolutionized how molecular biology studies are conducted. Its decreasing cost and increasing throughput permit profiling of genomic, transcriptomic, and epigenomic features for a wide range of applications. Microfluidics has been proven to be highly complementary to NGS technology with its unique capabilities for handling small volumes of samples and providing platforms for automation, integration, and multiplexing. In this article, we review recent progress on applying microfluidics to facilitate genome-wide studies. We emphasize on several technical aspects of NGS and how they benefit from coupling with microfluidic technology. We also summarize recent efforts on developing microfluidic technology for genomic, transcriptomic, and epigenomic studies, with emphasis on single cell analysis. We envision rapid growth in these directions, driven by the needs for testing scarce primary cell samples from patients in the context of precision medicine.

## I. INTRODUCTION

Next-generation sequencing (NGS) is transforming understanding of molecular biology at the genome scale. Since the completion of the human genome project in 2003, the cost of sequencing has significantly decreased, making it accessible to a large community of researchers. Advances in sequencing technologies require development of new sample processing procedures that complement NGS processes. Genomics and transcriptomics (i.e., study of the complete set of DNA or RNA of cells at a specific stage) have already achieved single cell sensitivity using conventional tube-based approaches.^1–6^ However, tube-based approaches were limited in their power of generating a large amount of NGS data, due to amplification bias and throughput (only up to tens of samples each batch at most). Such methods are not suitable for investigating the heterogeneity among single cells due to manual handling errors and the large number of experimental subjects. Epigenomics is the study of heritable modifications on DNA or histones without changes in the DNA sequence. It is an emerging field that usually requires a large amount of starting material for genome-wide examination (e.g., ChIP-seq, MeDIP-seq, and Bisulfite-seq). Benchtop versions of these assays usually do not provide an efficient way to test scarce cell samples from small lab animals and patients.

Microfluidics, which facilitates manipulation of liquid or suspension with extremely small volumes (pico to nanoliters), has gained wide popularity for examining tiny quantities of cell samples (down to single cells)^7–11^ and creating highly controlled microenvironments.^12–16^ Utilizing parallel structures, microfluidic devices are capable of processing hundreds of samples simultaneously within isolated tiny chambers. The miniaturized structures improve throughput and reduce reagent consumption, and the required amount of analytes.

In this review, we will discuss microfluidic technologies for genome-wide analysis with emphases on genomics, transcriptomics, and epigenomics. Proteomics and single cell analysis technologies that do not involve NGS will not be discussed here.^2,17–25^

## II. THE BASICS OF NEXT GENERATION SEQUENCING

**FIG. 1.**
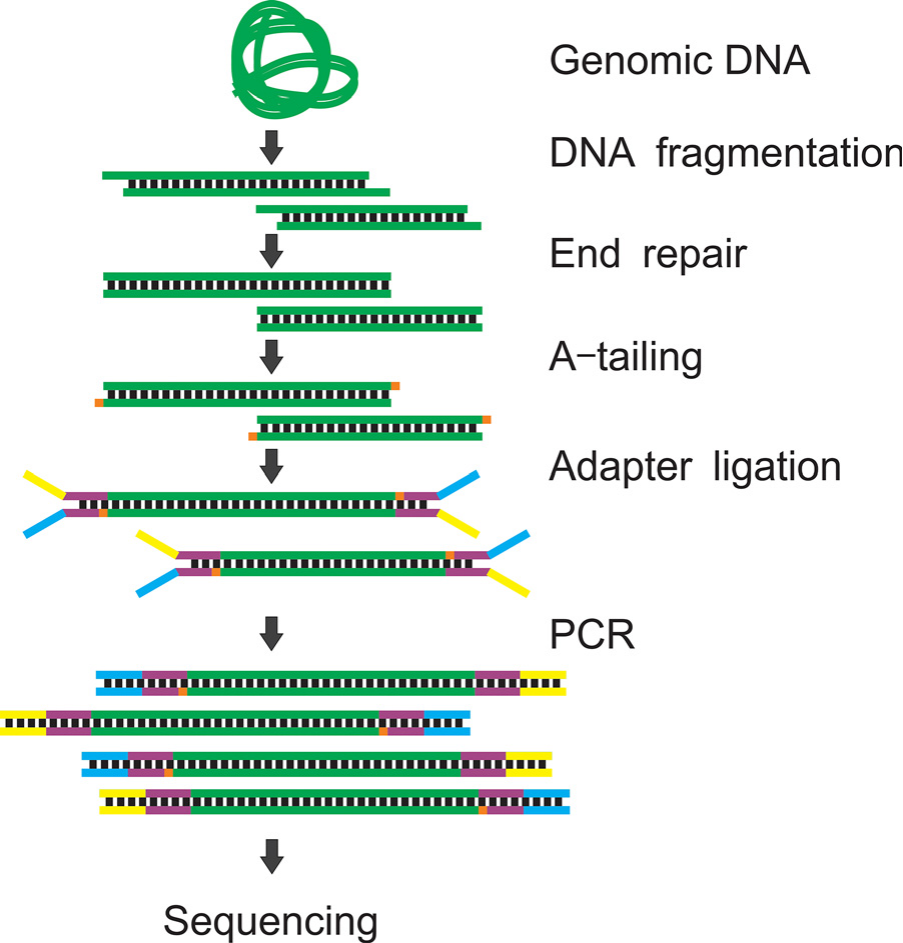
The NGS library preparation procedure. Genomic DNA is fragmented to 100–500 bp by sonication or enzyme digestion. The fragmented DNA is end-repaired to generate a blunt end. An additional dAMP is incorporated into the 3′ end of a blunt DNA fragment. The Y-shaped adapter is ligated to both ends of DNA. The ligated DNA is amplified by PCR to generate enough materials for sequencing.

There are a variety of NGS technologies based on different principles.^26^ In most NGS schemes (most notably sequencing by synthesis such as Illumina sequencing^27^), DNA synthesis is catalyzed by polymerase to add fluorescently labelled dNTPs onto the DNA templates during a series of cycles. At the end of each cycle, the fluorescent signals are analyzed to identify the added nucleotides. NGS allows processing millions of fragments in parallel, which significantly improves throughput and decreases sequencing costs. There are five major steps to prepare a sequencing sample/library: DNA fragmentation, end-repair/A-tailing, adapter ligation, amplification, and quality control/sample pooling (Fig. 1).

The starting material for library preparation needs to be fragmented to ∼100–500 bp to be properly amplified in the flow cell. The fragmentation is typically performed by sonication, enzyme digestion, or tagmentation. Next, a pair of sequencing adapters are ligated to both ends of the DNA. The ligated fragments attach to the flow cell by hybridization during sequencing. To maximize the ligation efficiency, the DNA fragments are usually subjected to end-repair and A tailing (generate adenine overhang to the 3′ end of DNA) before ligation. Depending on the amount of starting materials, the ligated products may be insufficient for sequencing (requiring ≥2 nM). Polymerase chain reaction (PCR) is the most commonly used approach to amplify the ligated products and generate enough materials for sequencing. The fragment sizes of amplified products are checked by gel electrophoresis or a Bioanalyzer to ensure that they are properly ligated. The libraries are then pooled to the desired concentration (2–10 nM) and ready for sequencing. Depending on the applications, the library preparation procedure may be different. We will discuss various sequencing-related procedures for the three major categories of applications, genomics, transcriptomics, and epigenomics below.

## III. GENOMICS

Next generation sequencing (NGS) conventionally requires nanograms or micrograms of DNA for library construction. New kits from various vendors (ThruPLEX DNA-seq Kit from Rubicon Genomics, NEBNext Ultra DNA Library Prep Kit from New England Biolabs, DNA SMART ChIP-Seq Kit from Clontech, PureGenome Low Input NGS Library Construction Kit from EMD Millipore, and Accel-NGS 2S Plus DNA Library Kit from Swift Biosciences) have reduced the starting material amount for library construction to 10 pg–1 ng.

When the amount of genomic DNA is scarce (e.g., in the case of single cell sequencing with 5–7 pg DNA from each cell), whole genome amplification (WGA)^28^ may be necessary. Earlier WGA approaches (primer extension preamplification (PEP)^29–31^ and degenerate oligonucleotide primed-polymerase chain reaction (DOP-PCR)^32^) were developed more than 20 years ago.^30,32^ Over the years, several more WGA methods have been developed and improved,^33^ including multiple displacement amplification (MDA),^34–37^ OmniPlex/GenomePLEX,^38^ PicoPLEX,^39,40^ and multiple annealing and looping based amplification cycles (MALBAC).^41,42^ These protocols improved the sensitivity and fidelity of WGA. In recent years, linear amplification based MDA has arguably become the most popular and successful WGA in terms of sensitivity, accuracy, and reliability.^33,43,44^ MALBAC was recently developed and showed promising results compared with MDA in terms of uniformity in amplification of various genes.^45–47^ Most of these protocols have been successfully applied on microfluidic platforms^46,48–55^ and some provided superior genome coverage and sequencing uniformity compared to benchtop versions.^46,48,52–54^

### A. Digital PCR

**FIG. 2.**
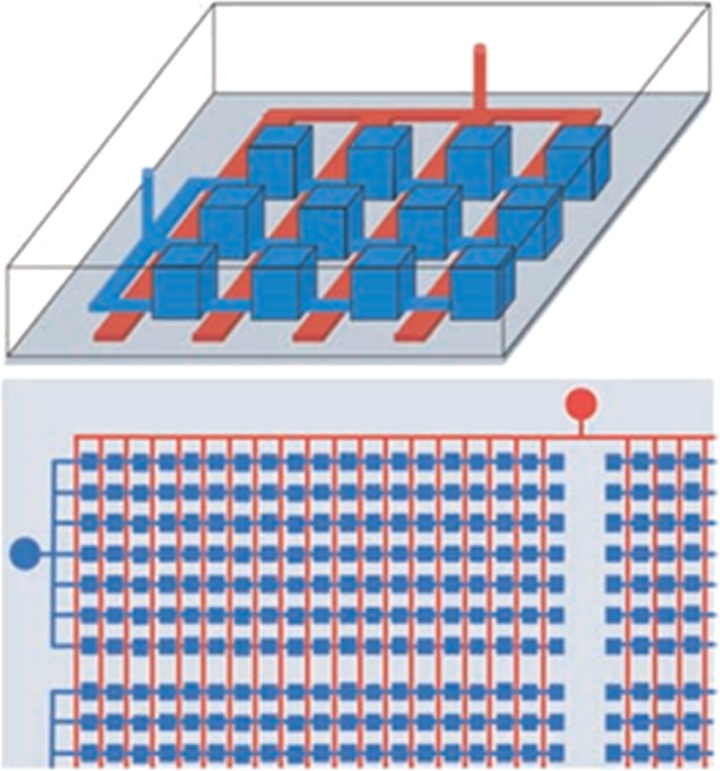
Microfluidic devices for Digital PCR. The top schematic diagram shows that the parallel chambers (blue) can be reversibly isolated by applying pneumatic or hydraulic pressure to the control channel network (red). The bottom schematic shows that a single valve connection is used to partition thousands of chambers. Reprinted with permission from Ottesen *et al*., Science **314**(5804), 1464–1467 (2006). Copyright 2006 American Association for the Advancement of Science.

Digital PCR is the first WGA application on a microfluidic platform (Fig. 2). Ottesen *et al*. utilized microfluidic digital PCR to study the heterogeneity of bacteria.^56^ DNA from bacteria of mixed species was diluted and loaded on a microfluidic chip to make sure only one or none (digitalized) gene sequence was contained in each PCR chamber. Primers designed for “all-bacterial” 16S rRNA gene were used for amplification. The digitalized amplification products were retrieved, re-amplified, cloned, and sequenced for analyzing the bacterial species. Similarly, digital PCR was used to study the host-virus interactions for individual bacteria.^57^ The small subunit (SSU) rRNA gene encoded by bacteria was amplified by “all-bacterial” primers, while viruses were amplified by degenerate primers. The two amplifications, labelled by two different fluorescent probes, were conducted simultaneously to discover the genuine bacteria-virus interaction.^57^ Although the digital PCR enabled the analysis of hundreds of cells simultaneously, the inherence of PCR needs pre-designed “broad-specificity” primers which limits its applications to a part of a simple genome (bacteria and virus) instead of the entire genome.

### B. Multiple displacement amplification

Multiple displacement amplification (MDA)^35–37^ is a non-PCR based isothermal amplification method which was applied first for WGA by Dean *et al*.^37^ MDA uses random exonuclease-resistant primers and strand displacing φ29 polymerase to amplify femtograms or picograms of DNA templates with length greater than 10 kb.^37^ The φ29 polymerase extends random primed hexamers until it reaches a newly synthesized DNA and then displaces the DNA strand and keeps polymerizing. The φ29 polymerase has an inherent 3′ to 5′ proofreading exonuclease activity which results in low error (1 in 10^6^−10^7^ bases^58^) and high fidelity of the amplification. MDA also generates a significant amount of DNA product, which is almost an unlimited source for genotyping or sequencing library preparation. It takes about 3 h to produce 1–2 *μ*g of DNA from a single cell. Several commercially available kits (REPLI-g from Qiagen and GenomiPhi V2 from GE Healthcare) have been widely used for many species.^59,60^ Due to the isothermal reactivity of MDA, it can be easily integrated onto microfluidic chips. There are four major formats of microfluidic MDA: micro-chamber, droplet, micro-well, and gel.

#### 1. Micro-chamber MDA

**FIG. 3.**
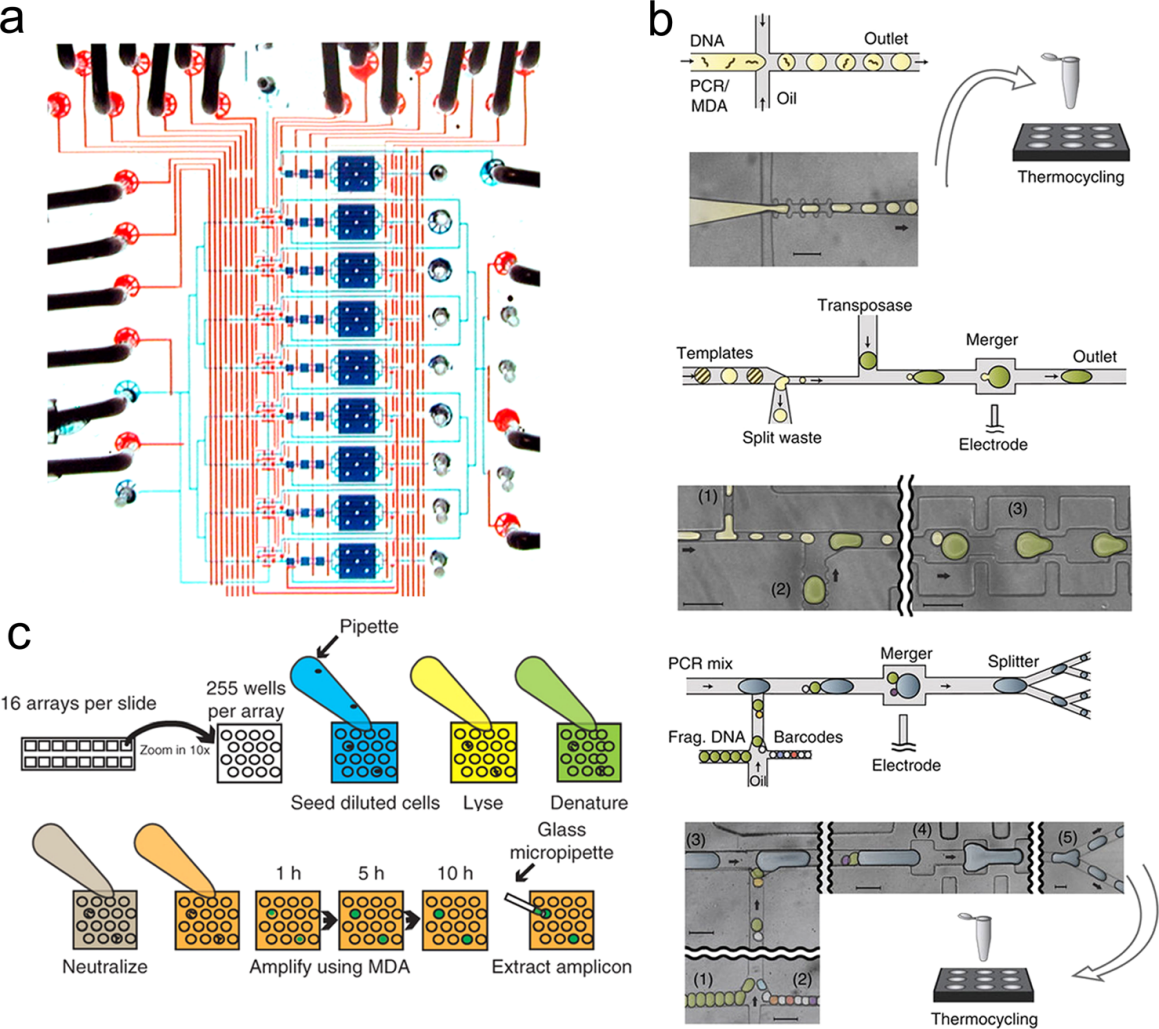
Microfluidic devices for MDA. (a) Micro-chamber MDA. A photograph of the single cell isolation and amplification chip. The fluidic chamber and channels are filled with blue dye and the control lines were filled with red dyes. Reprinted with permission from Marcy *et al*., PLoS Genet. **3**(9), 1702–1708 (2007). Copyright 2007 PLOS. (b) Droplet MDA. The single cell is lysed in a tube and mixed with MDA reagents. The mixture is either directly used for conventional MDA or used to generate droplets in a microfluidic device. Reprinted with permission from Lan *et al*., Nat. Commun. **7**, 11784 (2016). Copyright 2016 Macmillan Publishers Ltd. (c) Micro-well MDA (MIDAS). Each MIDAS chip contains 16 arrays of 255 micro-wells. The diluted cells are loaded in the microwells. Lysis solution, denaturing buffer, neutralization buffer, and MDA master mix are added to the microwells in sequence. The amplification procedure is monitored based on the growth of fluorescence. The amplicons are extracted for sequencing library construction. Reprinted with permission from Gole *et al*., Nat. Biotechnol. **31**(12), 1126–1132 (2013). Copyright 2013 Macmillan Publishers Ltd.

Marcy *et al*. first applied MDA for amplifying single microbial cell in a series of microfluidic chambers^48,49^ (Fig. 3(a)). Taking advantage of more than 20 pneumatic valves, they applied the REPLI-g MDA kit (Qiagen) on a PDMS (Polydimethylsiloxane)/glass chip. The chip integrated cell sorting, lysis, neutralization, and MDA amplification. This protocol was also used for amplifying DNA from ammonia-oxidizing archaea^61^ and redesigned for parallel amplification of 48 single sperm cells.^62^ Blainey and Quake^63^ applied digital DNA for enumeration of nucleic acid contamination, which was observed in previous work.^48,49^ Similarly, this method was modified for haplotyping of single cells.^64^ An additional chamber was designed to capture and lyse the individual metaphase cell for MDA. It was also adapted to the C1 integrated fluidics circuit (IFC) platform by Fluidigm.^50,51^ This automated sample preparation system not only improved the genomic coverage (∼90%),^50^ but also significantly increased the assay throughput^51^ (up to 96 single cells to be amplified and sequenced in parallel). It is worth noting that the small reaction volume (∼60 nl) in micro-chambers improved the uniformity of MDA compared to conducting MDA in 50 *μ*l bulk.^48^

#### 2. Droplet MDA

Droplet microfluidics provides a convenient way to generate reaction volumes of picoliters that help improve on genome recovery and amplification bias.^65^ MDA reagents and genomic DNA were dispersed in droplets suspended in oil. The emulsion MDA (eMDA) developed by Fu *et al*.^46^ showed a lower copy number variation compared to MALBAC and MDA. In eMDA, DNA fragments were distributed into the droplet. Each droplet contained one fragment (∼10 kb) on average. The droplets were collected in a micro-centrifuge tube and amplified for 8–10 h. The careful adjustment of fragment concentration improved the WGA uniformity and suppressed non-specific amplification. Nishikawa *et al*.,^66^ Sidore *et al*.,^67^ and Rhee *et al*.^68^ generated similar results to those of eMDA. Due to the compartmentalization, DNA molecules in each droplet were amplified to saturation and yielded high uniformity compared to bulk MDA. Lan *et al*. showed an interesting demonstration, single-molecule droplet barcoding (SMDB), which incorporated DNA barcoding technology with WGA^69^ (Fig. 3(b)). Single DNA templates are encapsulated in each droplet and amplified by MDA or PCR. The droplets containing Nextera transposase are then merged with droplets containing amplified products to fragment the DNA and add adaptors to both ends of the DNA fragments. Droplets of barcodes, PCR mix, and fragmented DNA were merged and subjected to PCR amplification. The barcodes were added onto the DNA templates during PCR. This protocol utilized multiple microfluidic devices to perform most of the steps, including template amplification, fragmentation, and barcoding. The barcodes allowed unique tagging of all reads from the same template and obtained synthetic read-lengths up to 10 kb in length.

#### 3. Micro-well MDA

Gole *et al*. developed the micro-well MDA system (MIDAS)^52^ (Fig. 3(c)). The MIDAS contains 4080 micro-wells, each with a volume of 12 nl, on a single chip. The cells were randomly distributed into micro-wells. The cell lysis, denaturation, neutralization, amplification, and amplicon extraction were performed by pipetting without any micro-valves and this dramatically simplified the system. More importantly, MIDAS recovered more than 98% of the *E. coli* genome at 1× coverage. It is 50% more compared to previously published results.^52^ For sperm cells and neuronal nucleus, MIDAS shows much lower amplification bias compared to many other protocols, including MALBAC, in-tube MDA, and microfluidic MDA.

#### 4. In-gel digital MDA

Conventional single cell MDA requires discrete physical boundaries. Xu *et al*. developed a hydrogel-based virtual microfluidics for MDA.^53^ It relied on hydrogel-limited diffusion to compartmentalize templates and reaction products. It is an alternative to the complicated microfluidic system for compartmentalization.^53^ They covalently crosslinked poly(ethylene glycol) (PEG) hydrogels under mild conditions which did not damage templates or inhibit subsequent reactions. The mesh size of gels was about 25 nm and allowed diffusion of oligo primers and polymerase. Cells and genomic DNA were retained in the gel because of their large sizes. This approach was applied to purified DNA or cultured bacteria. The bacterial cells were embedded in the gel and lysed by enzymatic and heat treatment. MDA reagents were introduced by diffusion. After amplification for ∼8 h, the gel punches were manually recovered and reamplified. Under 20× mean mapping depth, 30%–60% of the genome was recovered from a single cell sample. It showed improved 5× lower chimeric reads compared to in-tube MDA which benefited the analysis of rearrangements and mapped read counting.

### C. Quasilinear amplification

MALBAC^41,42^ is the most recently developed quasilinear WGA method. It combines linear and exponential amplification (PCR). The specially designed primer contains 8 variable nucleotides and 27 common nucleotides. During linear amplification, the 8 variable nucleotides randomly bind to the genomic DNA. After extension, the common nucleotide sequence attaches to only one end of the amplicon (semi-amplicon). After a second round of priming and extension, the semi-amplicons are extended to full amplicons, which have a common nucleotide sequence on both ends. The full amplicon loop prevents them from being further amplified in the following amplification cycles. This leads to an almost linear amplification and uniform genome coverage. In the exponential amplification, the loops of the amplicons are opened and amplified by regular PCR. PicoPLEX,^39^ developed by Rubicon Genomics, is very similar to the MALBAC protocol. Both cases utilize quasi-random priming, linear amplification, and exponential amplification.

#### 1. PicoPLEX in droplet

**FIG. 4.**
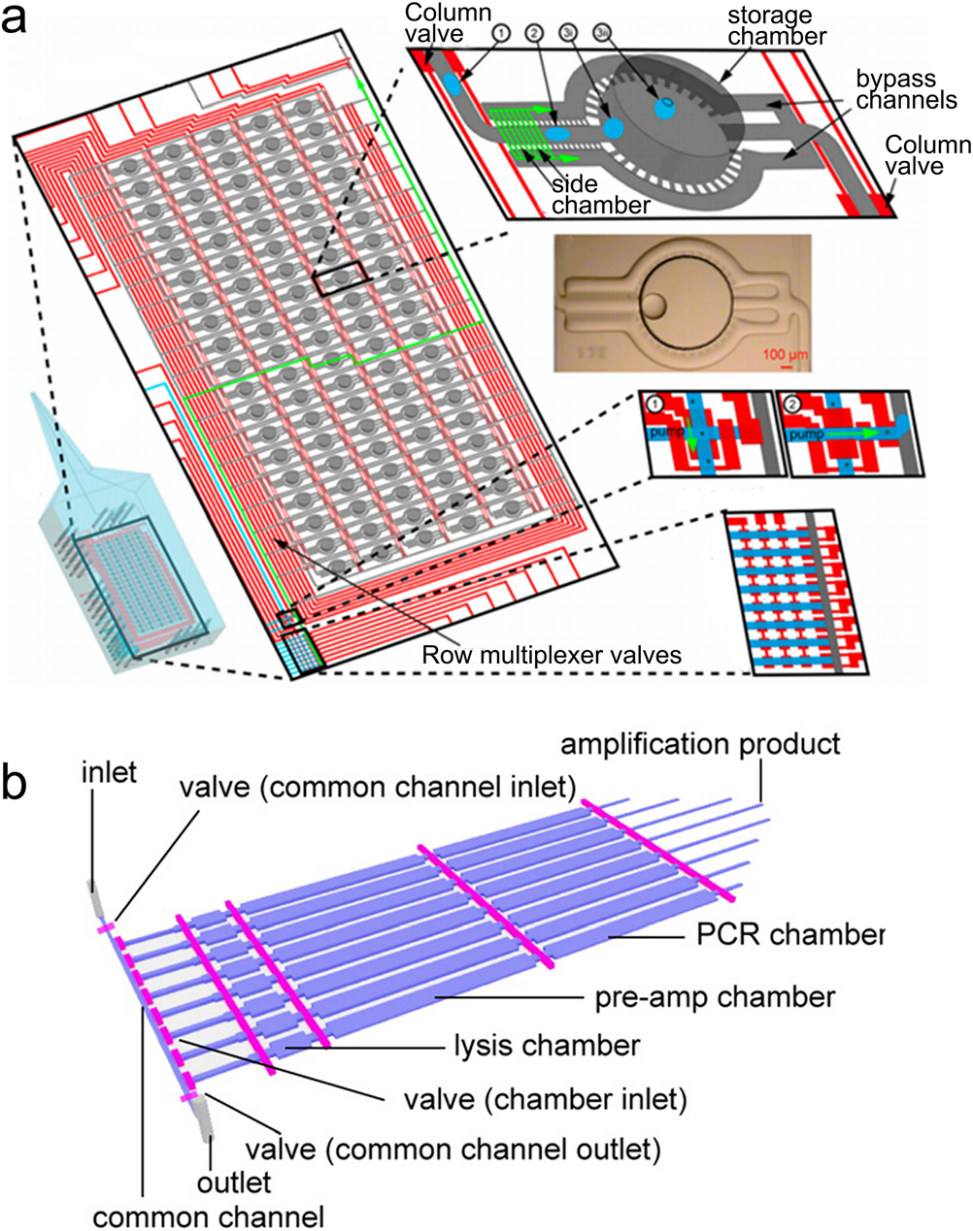
Microfluidic devices for quasilinear amplification. (a) The programmable microfluidic reaction array using PicoPLEX. Addressable array of 19 × 5 storage chambers that are controlled by valves (red). Reprinted with permission from Leung *et al*., Proc. Natl. Acad. Sci. U.S.A. **109**(20), 7665–7670 (2012). Copyright 2012 National Academy of Sciences. (b) Microfluidic MALBAC. Reprinted with permission from Yu *et al*., Anal. Chem. **86**(19), 9386–9390 (2014). Copyright 2014 American Chemical Society.

Leung *et al*. designed a droplet-based microfluidic device to analyze single microbes^54^ (Fig. 4(a)). This versatile device consisted of 95 individual nanoliter-chambers that allows cell sorting, cultivation, qPCR, and WGA. They implemented the multi-step PicoPLEX WGA protocol by merging multiple droplets. Their single cell reactions with the highest coverage were comparable to the bulk reaction with 1000 cells.

#### 2. Microfluidic MALBAC

Yu *et al*. designed a PDMS microfluidic device for paralleled single mouse embryonic stem (mES) cell MALBAC^55^ (Fig. 4(b)). The chip contained a series of chambers (lysis, pre-amplification, and PCR) which were very similar to the micro-chamber MDA chip.^48,49^ Single cells were trapped in each chamber. The cells were then transferred to a lysis chamber (75 nl) using lysis buffer containing protease. The lysis step took 90 min at 50 °C and was heat inactivated at 75 °C for 20 min. The preamplification buffer was injected to fill both the lysis chamber and preamplification chamber (500 nl). After 10 cycles of MALBAC preamplification, samples were mixed with PCR buffer and filled the additional PCR chamber (500 nl). After 16 cycles of PCR, each single cell generally yielded 50 ng DNA. The on-chip MALBAC (∼2.4%) showed a lower contamination level than conventional MALBAC (4.8%). The raw sequencing data showed significant variation in the coverage depth. The uniformity was improved when the data were normalized based on GC content (guanine-cytosine content). It indicated that MALBAC amplification favors GC rich regions.

### D. Targeted sequencing

Efforts have been made to develop “targeted sequencing” methods, in which genomic regions are selected before sequencing to lower the cost.^70–74^ The selected regions are sequenced with considerably lower cost than whole-genome sequencing. There are three widely used approaches for target enrichment: PCR, molecular inversion probes (MIPs), and hybrid capture.

#### 1. PCR

PCR has been widely used for sample enrichment. ThunderStorm platform, developed by RainDance Technologies, uses microdroplets to conduct millions of PCR in parallel.^73,75^ The system integrates droplet generation and droplet merging on a single microfluidic device. Each droplet contains only one set of primers to eliminate the difficulty with multiplexed PCR. The droplets are processed in a tube for PCR and then coalesced for sequencing. Several primer panels are currently available from the company.

Forshew *et al*. used a microfluidic system (Access Array, Fluidigm) to perform parallel single-plex amplification from multiple preamplified samples using multiple primer sets.^76^ They designed a set of 48 primer pairs to amplify 5995 bases of genomic sequences. The Access Array system allows up to 48 samples per run with 50 ng of input DNA.

Eastburn *et al*. developed MESA (Microfluidic droplet Enrichment for Sequence Analysis) for isolating genomic DNA fragments in microfluidic droplets and performing TaqMan PCR reactions to identify droplets containing a desired target sequence. PCR reagents, TaqMan primers/probes, and genomic DNA were encapsulated in microdroplets. The droplets were collected and subjected to PCR amplification. The droplets were sorted based on fluorescence at a rate of 1 kHz on chip and positive droplets are recovered. The TaqMan amplicons were then enzymatically removed before sequencing. Using this technology, they reached averagely 94.87% alignment rate and 84.71% uniquely alignment rate.

#### 2. Molecular inversion probes

Molecular inversion probes (MIPs) are based on target circularization. Single stranded oligonucleotides consist of a common liner flanked by target-specific sequences.^77^ The oligonucleotides anneal to the target and become circularized by ligase. The circularized species are PCR amplified using primers targeting a common linker, and uncircularized species are digested by exonucleases. Although MIPs have been utilized for target selection in a microfluidic chip,^78^ next generation sequencing has not been involved.

#### 3. Hybrid capture

Another major strategy to capture target sequences is using hybridization. Depending on the reaction phase, hybrid capture is divided into two categories: on-array capture and in-solution capture. For on-array capture, DNA fragments are hybridized to immobilized probes by matching their sequences. Non-targeted fragments are washed away and targeted fragments are then denatured and eluted.^79,80^ For in-solution capture, the hybridization happens in solution instead of on the surface of solid phase. The hybridized molecules are then collected by beads that target biotin-labeled probes.^81^ Roche NimbleGen, Agilent, Febit, and Illumina have announced their microarray products, which contain millions of probes. The performance of these products has been extensively reviewed.^82,83^

## IV. TRANSCRIPTOMICS

The transcriptome is the complete set of RNA in the cell at a specific stage. The aim of transcriptomics is to identify and quantify all the RNA. In early transcriptomic studies, PCR was the major tool for RNA quantification. Microfluidic qPCR has been used for integrated mRNA quantification over 15 years.^84–92^ PCR only allows the measurement of a few loci for each sample. In contrast, genome-wide approaches, such as RNA-seq and microarrays, are commonly used for transcriptomics these days. These approaches allowed analyzing the expression of more than 20 000 genes simultaneously. Due to the decreased cost and lowered bias, RNA-seq is becoming the most popular approach. There are a number of protocols that have been developed for RNA-seq, down to single cells, including T7 based linear amplification,^93,94^ template switching, CEL-seq,^95^ Quartz-seq,^96^ WGA-based methods,^97,98^ and barcoding-based methods.^99–103^ The benchtop RNA-seq protocols have previously been reviewed.^2,4,104,105^ Among those protocols, T7-based linear amplification, template switching, and barcoding-based methods have been adapted to a microfluidic platform and will be discussed here.

### A. T7-based linear amplification

**FIG. 5.**
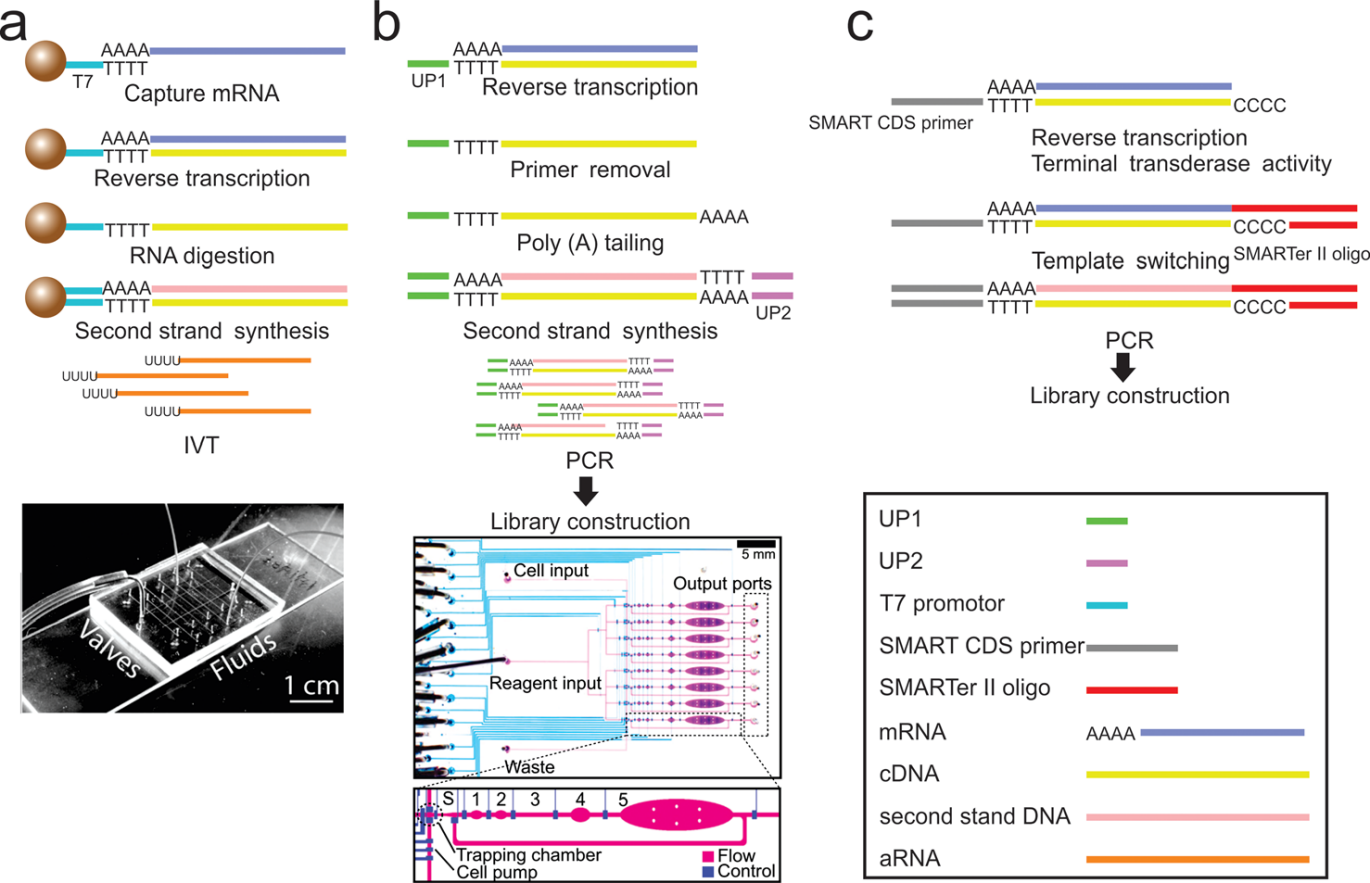
Representative RNA-seq protocols and the corresponding microfluidic devices. (a) T7 based linear amplification and the corresponding microfluidic device. (b) Single cell RNA-seq based on T7 linear amplification and the corresponding microfluidic device. (c) The mechanism of SMART-seq. (a) Reprinted with permission from Kralj *et al*., Lab Chip **9**(7), 917–924 (2009). Copyright 2009 The Royal Society of Chemistry. (b) Reprinted with permission from Streets *et al*., Proc. Natl. Acad. Sci. U.S.A. **111**(19), 7048–7053 (2014). Copyright 2014 National Academy of Sciences.

T7 RNA polymerase, which permits a linear amplification of mRNA, is used for *in situ* transcription.^106^ It was the first protocol for global gene expression profiling conducted on a microfluidic platform^91^ (Fig. 5(a)). Functionalized microbeads are used to capture mRNA by hybridizing the poly-A tail. The RNA is reverse transcribed, followed by RNA digestion and second strand synthesis to generate double stranded cDNA (complementary DNA). The cDNA is amplified by *in vitro* transcription on-chip to generate aRNA (antisense RNA) for microarray analysis. This protocol requires 20 pg to 10 ng purified RNA and approaches single cell sensitivity.

Single cell gene expression measurement was first demonstrated by Eberwine *et al*., also based on linear amplification of T7 polymerase.^93^ Eberwine' s protocol was then improved by Tang *et al*. to generate cDNA as long as 3 kbp without bias^94^ (Fig. 5(b)). By combining with a SOLiD sequencing system, it detected 5270 more genes than a microarray. The mRNA is first reverse transcribed to cDNA using an oligo (dT) attached PCR primer (UP1). Following primer removal and attachment of the poly-A tail to the first strand cDNA, another PCR primer (UP2) with a poly-(dT) tail is used to synthesize the second strand. The cDNA with primers on both ends is amplified by PCR and then used for library construction. Streets *et al*. then adapted this protocol to a microfluidic platform^107^ (Fig. 5(b)). The chip consists of 8 units with 6 connected chambers in each unit. Single cells are trapped in the sorting chamber and then subjected to cell lysis, reverse transcription, poly-A tailing, primer digestion, and second strand synthesis in the following chambers. They investigated cell heterogeneity by sequencing 56 single mouse Embryonic Stem cells (mES cells).

### B. Template switching

Another major type of RNA-seq protocol is based on utilizing the template-switching site located preferentially at the 5′ end of the mRNA by Schmidt and Mueller.^108^ Islam *et al*. described a single-cell tagged reverse transcription (STRT) protocol.^109^ mRNA is reverse transcribed into cDNA with 3–6 added cytosines (terminal transferase activity). A helper oligo introduces a barcode and a primer sequence into cDNA by template switching. The product is then amplified by single-primer PCR, immobilized on beads, fragmented, and A-tailed. Sequencing adapter P1 is ligated to the free end of the product, and adapter P2 is introduced by PCR with a primer tailed with a P1 sequence.

Smart-seq, which is the most popular single cell mRNA-seq protocol, also utilizes the template switching mechanism^110^ (Fig. 5(c)). It allows coverage across the full-length transcripts (not achievable by STRT). Smart-seq2^111^ improved the performance over the original SMART-seq in terms of yield and length of cDNA libraries. For Smart-seq, cells are lysed in reverse transcription compatible solution. The mRNA is reverse transcribed by an oligo-dT containing primer, and the reaction is stopped by adding un-templated C nucleotides followed by template switching. cDNA is then sheared by a Covaris or transposase tagmentation based protocol to generate sequencing libraries. The Smart-seq protocol was adapted to the C1 platform from Fluidigm for high throughput single cell RNA-seq. Single cells are captured and lysed on-chip. The RNA is reverse transcribed, pre-amplified, and eluted. The elution is amplified off-chip by PCR to generate enough materials for sequencing. Up to 384 single-cell samples are pooled together for a single sequencing reaction. It has been used to investigate heterogeneity in various tissues, such as brain^112,113^ and bone marrow derived dendritic cells.^114^

### C. Barcoded mRNA capture beads

For profiling mRNA from thousands of cells, microfluidics provides a unique high-throughput platform. To distinguish each single cell, barcoded beads are introduced into the system. The barcoding process can be performed in either micro-wells^99,100^ or droplets.^101–103^ The beads usually contain a T7 promoter or PCR handle, cell barcodes, molecular barcodes (UMIs), and a poly-T tail. The T7 promotor or PCR handle is the sequence that can be recognized by polymerases. Cell barcodes are used to identify each single cell. UMIs are designed to recognize the unique RNA sequence in each cell. The duplicated reads can be filtered based on the UMIs to reduce amplification artifacts. The poly-T tail is used to capture mRNA containing poly-A sequences.

**FIG. 6.**
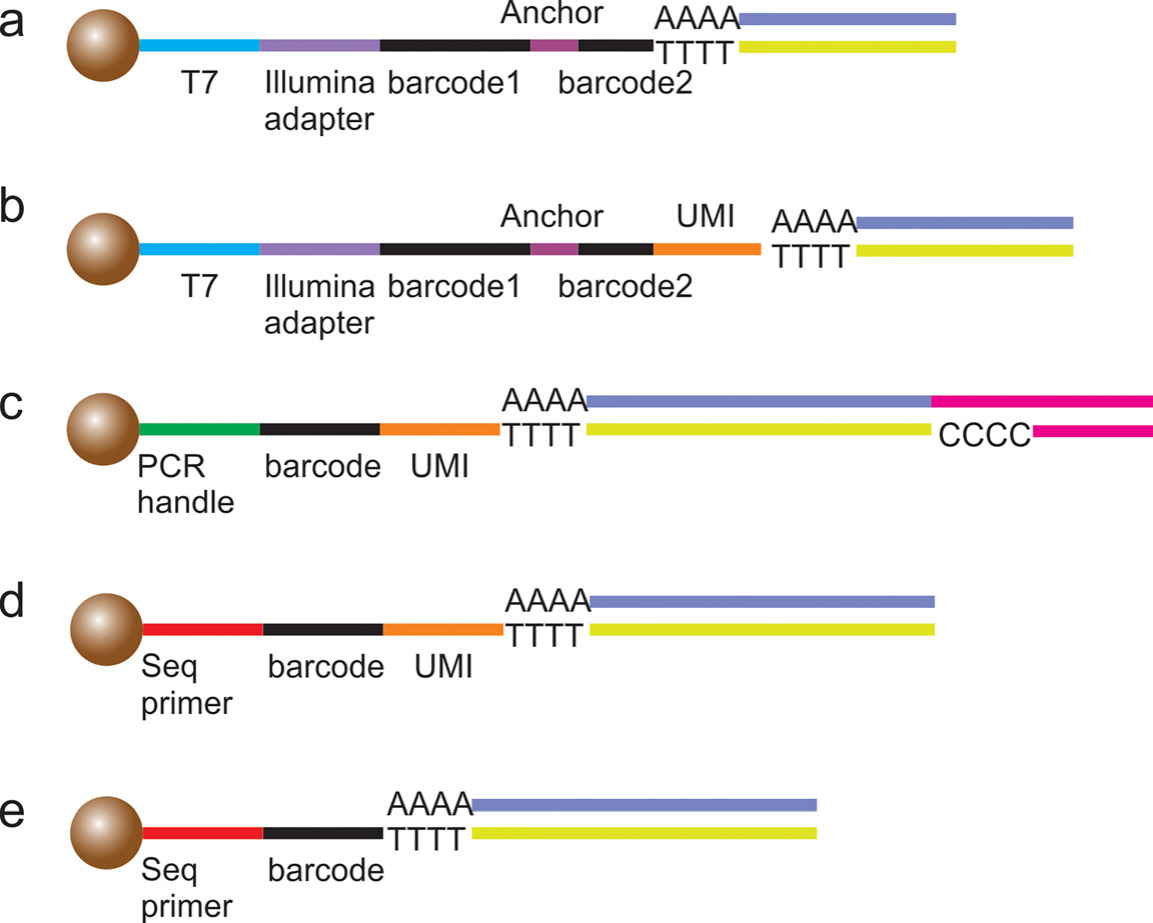
Five kinds of barcoded beads for RNA sequencing. mRNA is captured by ploy-T tail and reverse transcribed. (a) The bead structure was adapted from CEL-seq.^95^ Each cell was barcoded before linear amplification. (b) UMI was incorporated to reduce amplification artifacts. (c) The beads used in the Drop-seq protocol which took advantage of template switching.^103^ (d) The beads used by Fan *et al*. to generate sequencing-ready libraries. (e) The beads used in the Hi-SCL protocol.^101^

There are five currently available protocols (Fig. 6). Bose *et al*.^100^ adapted their protocol from CEL-seq,^95^ which barcoded each cell before the linear amplification (Fig. 6(a)). Klein *et al*. incorporated the UMIs into the bead sequences. By analyzing UMIs, the duplicated information is filtered to reduce the amplification artifacts^102^ (Fig. 6(b)). Macosko *et al*.^103^ developed the Drop-seq protocol, which took advantage of the template switching used in the STRT protocol^109^ (Fig. 6(c)). Drop-seq is based on template switching and PCR instead of T7-based linear amplification. Fan *et al*. used multiple rounds of PCR to directly generate sequencing-ready libraries. Unfortunately, the usage of gene-specific primers may limit its application^99^ (Fig. 6(d)). The Hi-SCL protocol, developed by Rotem *et al*., showed the simplest barcoding sequences on beads, which only consisted of sequencing primer, barcode, and poly-T tail. However, the experimental setup was the most complicated among the five protocols^101^ (Fig. 6(e)).

## V. SIMULTANEOUS SEQUENCING OF DNA AND RNA

**FIG. 7.**
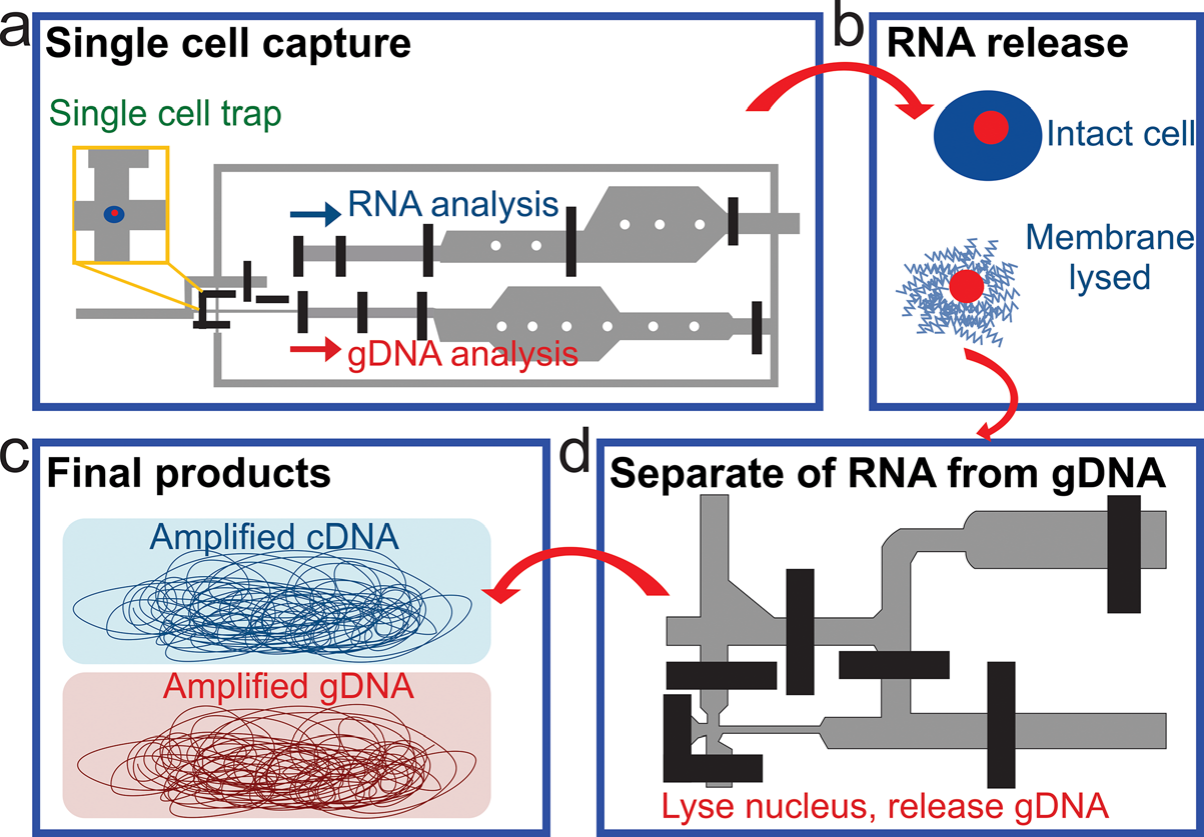
A microfluidic device for DNA and RNA analysis from the same cell. Schematics of the process flow. (a) A single cell is trapped in a small chamber. (b) The captured single cell is lysed by the membrane-selective lysis buffer. (c) RNA is separated from the intact nucleus and reverse transcribed to cDNA. (d) cDNA and gDNA are subjected to whole pool amplification. Reprinted with permission from Han *et al*., Sci. Rep. **4**, 6485 (2014). Copyright 2014 Macmillan Publishers Ltd.

Simultaneous sequencing of genome, transcriptome, and epigenome in the same single cell provides fresh and rare insights into how these molecular programs interact with each other during biological processes. Such measurement reveals correlations among these variations and helps understand the heterogeneity in the cell population. The most critical step of simultaneously sequencing RNA and DNA is to separate RNA from DNA. The most common way is to partially lyse single cells without breaking the nucleic membrane.^115,116^ The cytoplasmic mRNA is released and genomic DNA is still contained in the nuclei. The mRNA and gDNA (genomic DNA) are separated and used for RNA-seq and DNA-seq library preparation, respectively. Han *et al*. applied this approach in a parallel microfluidic device, which increased mRNA-to-cDNA conversion efficiency by ∼5 fold^117^ (Fig. 7). The single cells were trapped in a tine chamber (Fig. 7(a)) and the cells were partially lysed to release RNA (Fig. 7(b)). The released RNA was separated from the nucleus. Once separated, the nucleus was lysed and the gDNA was released (Fig. 7(c)). The gDNA and RNA were then amplified and sequenced. The drawback is that an important portion of the mRNA in the nuclei (nucleic mRNA) may not be completely released, which leads to the inaccuracy of the RNA-seq result.

To recover nucleic mRNA, Macaulay *et al*. designed a modified oligo-dT primer, which is attached to streptavidin coated magnetic beads. Cells are completely lysed, such that all mRNA and gDNA are released.^118^ The oligo-dT primer binds to the mRNA, and then the mRNA is physically separated. Dey *et al*. designed a quasilinear amplification strategy to quantify the gDNA and mRNA without physical separation.^119^ Due to the complexity of these protocols, they have not been conducted on microfluidic platforms.

## VI. EPIGENOMICS

Epigenomics concerns study of epigenetic modifications in the genome.^120^ Epigenetic modifications are reversible and heritable modifications on DNA or histones that do not change DNA sequences. The most common epigenetic modifications include histone modifications, DNA methylation, and non-coding RNA (ncRNA). These tags change chromatin structure and DNA accessibility thus regulating gene expressions. These modifications play critical roles in normal development and diseases, such as brain development^121^ and tumorigenesis.^122^ Unlike genomic analysis that mostly concerns only DNA sequence and amplification, most of the epigenomic assays require a large amount of starting chromatin or DNA due to steps other than PCR. For example, 1 × 10^6^ cells are required for histone modification profiling, and micrograms of DNA is needed for DNA methylation analysis. To overcome this drawback, several microfluidic based approaches^123–131^ have been developed and dramatically improved the sensitivity and throughput of conventional assays. Utilizing microfluidic technologies for epigenomic profiling is a fast growing field.

### A. Bisulfite conversion

In eukaryotic DNA, methylation often occurs at the cytosine to yield 5-methylcytosine (5-mC). DNA methylation represses gene expression by blocking promoters where the transcription factors (TFs) are supposed to bind to. Extensive studies have demonstrated that DNA methylation plays a major role in many processes, such as cellular proliferation, differentiation, and various diseases.^132,133^ Some studies have also indicated that there is connection between histone modification and DNA methylation at certain genomic loci.^134,135^

To examine the genome-wide DNA methylation profile, bisulfite conversion coupled with next generation sequencing is generally regarded as the gold standard. Bisulfite conversion enables methylation analysis at single base resolution.^136–138^ The method is based on the difference in reactivity of sodium bisulfite with unmethylated and methylated DNA. Sodium bisulfite converts unmethylated cytosines to uracils while methylated cytosines remain the same. After conversion, the original DNA methylation status is identified by quantitative PCR or sequencing. Bisulfite sequencing has been demonstrated at a single cell level.^139,140^ It can also be coupled with other sequencing technology (RNA-seq)^116,141^ to obtain more information from the same cell.

Efforts have been made to perform bisulfite conversion on a microfluidic chip. Shin *et al*. designed a droplet based platform for bisulfite conversion.^142^ DNA is bound to the surface of magnetic silica beads under low pH and high chaotropic salt concentration. The DNA can be released by reversing these conditions. By moving the DNA/bead complex, the DNA is transferred into different buffers for conversion and cleanup. As a comparison, Yoon *et al*.^143^ used a glass surface as the substrate to adsorb DNA instead of silica beads. The denatured DNA is first mixed with bisulfite cocktail and converted on chip. The converted DNA is mixed with guanidine hydrochloride and adsorbed to the glass surface by electrostatic interaction. In all these cases, converted DNA was detected by PCR for analyzing the methylation status. Some efforts have also been made to improve the accuracy and throughput of PCR using microfluidic devices.^144–147^ There has been no demonstration of whole-genome bisulfite sequencing using microfluidic devices.

### B. Affinity-based approaches

#### 1. Chromatin immunoprecipitation (ChIP)

In eukaryotic cells, DNA wraps around globular histone cores and forms nucleosomes. The nucleosomes pack together and form chromatins. The histone tails can be covalently modified, such as acetylation, methylation, phosphorylation, and ubiquitination. Acetylation and methylation are the most common modifications. Chromatin immunoprecipitation (ChIP) is the primary technology used to examine histone modifications.^148^ It can also be used to determine if a specific protein (i.e., transcription factors) interacts with the genome. ChIP is divided into two categories, depending on the method used to process the chromatin, XChIP and NChIP. For NChIP (native ChIP), chromatin is not cross-linked so that it is suitable for mapping histone marks or transcription factors that strongly bind to DNA. In the case of NChIP, the chromatin is fragmented by micrococcal nuclease (MNase) digestion to yield a ladder of DNA fragments corresponding to the size of multiple nucleosome cores plus the linker (∼170 bp). For XChIP (cross-linked ChIP), chromatin is stabilized by formaldehyde crosslinking, which makes it inaccessible to MNase digestion. Sonication is typically used for chromatin fragmentation in XChIP. The fragmented chromatin (by sonication or enzyme digestion) is selectively targeted by specific antibodies that are immobilized on the surface of magnetic or agarose beads. The chromatin/bead complexes formed by immunoprecipitation are washed, and then the enriched DNA is eluted and purified. The ChIPed DNA can be detected by quantitative PCR (ChIP-qPCR) for examining a few loci, NGS (ChIP-seq) or microarrays (ChIP-chip) for genome-wide profiling. Conventional ChIP assays require ∼1 × 10^6^ cells for ChIP-qPCR and ∼10 × 10^6^ cells for ChIP-seq.

##### a. ChIP-qPCR

The recent advances in microfluidic ChIP-qPCR were reviewed by Matsuoka *et al*.^149^ The first applications of ChIP on a microfluidic platform were independently developed by Oh *et al*.^123^ and Wu *et al*.^124^ Oh *et al*. designed a flat chamber (bead reservoir) to hold micro-agarose beads.^123^ The bead reservoir is connected to the dispersion channels via short and shallow channels, which stops microagarose beads while allowing solution to flow through. This chip design did not involve the use of complicated micro-valves. It requires 2.5 × 10^6^ cells as the starting material, which is similar to the performance of the conventional assay. Wu *et al*. designed AutoChIP^124^ and HTChIP^125^ for automated high throughtput ChIP-qPCR analysis using sheared chromatin corresponding to 1000–2000 cells for each sample. Chromatin is actively mixed with antibody-bead complexes in a circulating peristalic mixer. The chromatin-antibody-bead complexes are then stacked to a column by micromechanical valves and washed by buffers. They were able to perform ChIP assay for 4 and 16 samples simutaneously.

We developed microfluidic ChIP-qPCR assay based on 50 cells.^126^ This protocol utilizes N-ChIP (native chip) coupled with MNase digestion. Intact cells are directly loaded on chip instead of sonicated chromatin. The cells are lysed and digested by MNase. The fragmented chromatin is then forced to flow through a pre-packed IP bead bed that occupies a large fraction of the chamber in a connected micro-chamber. This technique allowed sensitive ChIP-qPCR with as few as 50 cells.

We also integrated sonication into a microfluidic chip for profiling histone modification and DNA methylation (i.e., MeDIP-qPCR).^127^ Chromatin or DNA is sheared by a transducer which is attached to the glass bottom of the chip. Compared to our previous protocol,^126^ the current approach works with cross-linked cells (X-ChIP) and this may extend the application to profile transcription factors. Cao' s work reached similar sensitivity (∼100 cells) to Geng' s work with a significantly improved signal-to-noise ratio (fold enrichment).

##### b. ChIP-seq

ChIP-qPCR assay only examines epigenetic modifications at a few loci in the genome. To extend the analysis to genome-wide profiling, recent efforts have been made toward production of substantial ChIP DNA for sequencing.

**FIG. 8.**
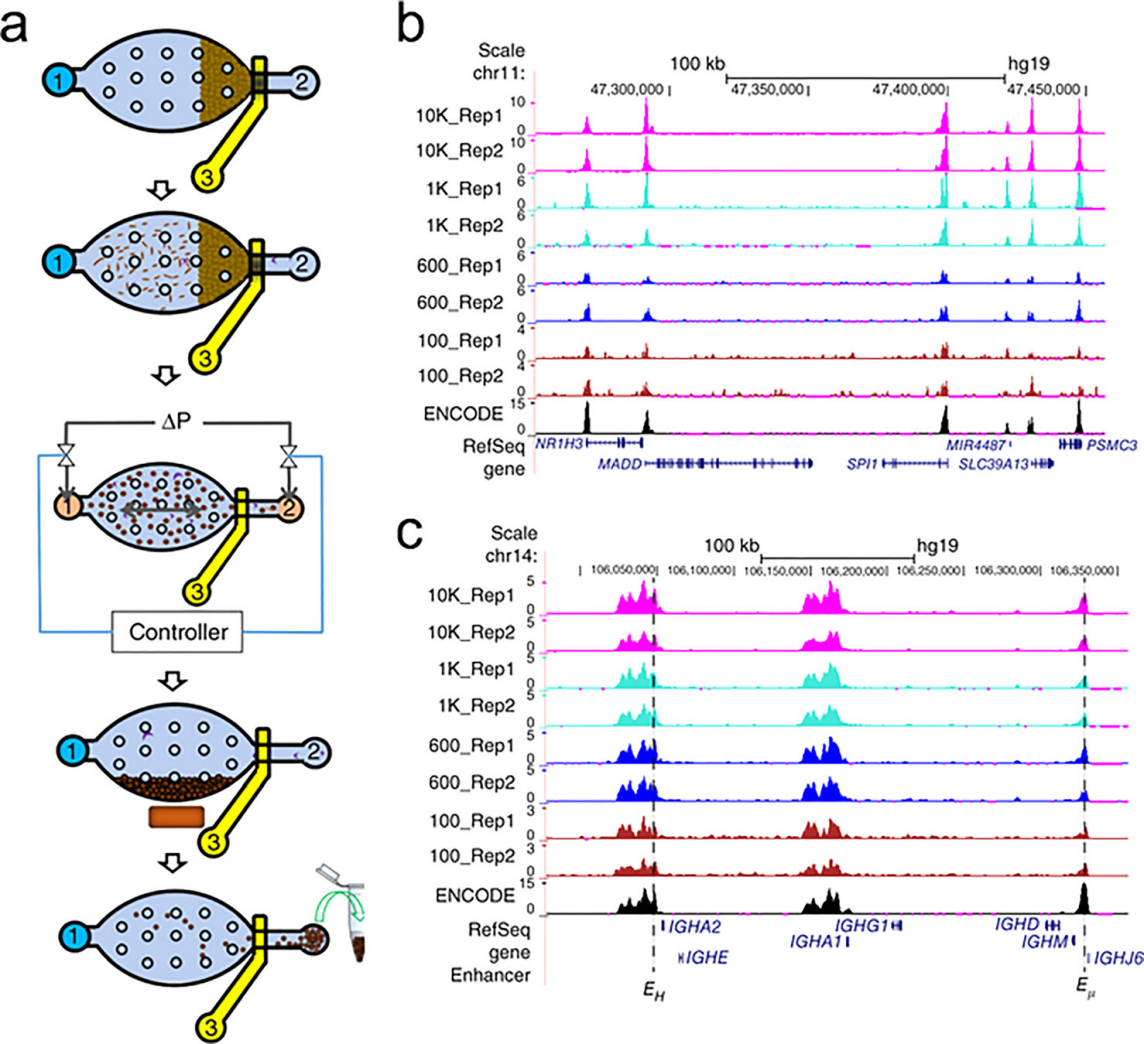
MOWChIP-seq for histone modification analysis. (a) Overview of the five major steps of MOWChIP-seq protocol: (i) formation of a packed bed of magnetic beads; (ii) chromatin is flowed through the packed bed for immunoprecipitation; (iii) oscillatory washing; (iv) removal of the unbound chromatin fragments and debris by flushing the chamber; (v) collection of the IP beads. (b) Normalized H3K4me3 MOWChIP-seq signals with various sample sizes. (c) Normalized H3K27ac MOWChIP-seq signals. Reprinted with permission from Cao *et al*., Nat. Methods **12**(10), 959–962 (2015). Copyright 2015 Macmillan Publishers Ltd.

We developed a microfluidic oscillatory washing–based ChIP-seq (MOWChIP-seq)^129^ protocol for profiling histone marks (H3K4me3 and H3K27ac) using as few as 100 cells (Fig. 8). In MOWChIP-seq, chromatin is flowed through a packed bed of antibody coated beads (Fig. 8(a)). The packed bed leads to high-efficiency adsorption but also increases nonspecific adsorption and physical trapping. The chromatin/bead complexes are washed by oscillatory washing in two buffers, which effectively removes non-specifically absorbed chromatins. The combination of packed bed adsorption and oscillatory washing was key to collection of ChIP DNA at the theoretical limit with high enrichment. Using as few as 100 cells, we generated a similar H3K4me3 and H3K27ac profile to published ENCODE datasets (Fig. 8(b)).

Shen *et al*. used a microfluidic chip to profile the H3K4me3 landscape.^128^ The chip shared similar structures to the ChIP-qPCR devices developed by Wu *et al*.^124,125^ The chromatin and beads are circulated in a dead-end flow channel for immunoprecipitation. The chromatin-antibody-bead complex is trapped to form a column and washed. This protocol was able to detect the histone mark from 1000 mouse early embryonic cells.

**FIG. 9.**
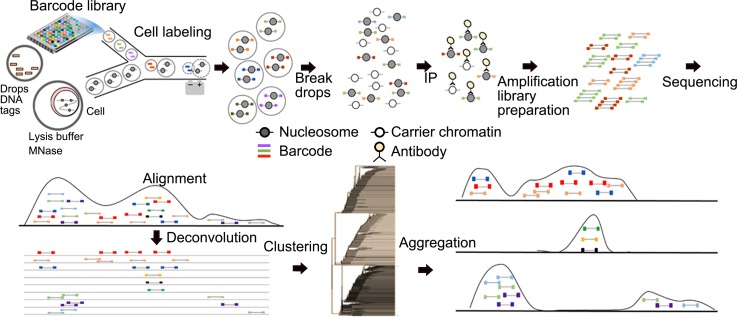
Drop-ChIP procedure for single cell ChIP-seq. Drops containing DNA barcodes are prepared by emulsifying DNA suspensions. Cells are encapsulated and lysed in drops, and then their chromatins were fragmented by MNase digestion. Chromatin-containing drops and barcode drops are merged in a microfluidic device, and DNA barcodes are ligated into chromatin fragments. Drops are combined and immunoprecipitated with “carrier” chromatin. The enriched DNA is sequenced. Sequencing reads are identified by their barcodes to generate single cell profiles (left) and then aggregated to produce subpopulation profiles (right). Reprinted with permission from Rotem *et al*., Nat. Biotechnol. **33**(11), 1165–1172 (2015). Copyright 2015 Macmillan Publishers Ltd.

Drop-ChIP protocol, developed by Rotem *et al*., collected ChIP-seq data at a single cell level^131^ (Fig. 9). Their strategy was to extract and digest chromatin of single cells in individual droplets. The fragmented chromatin is labeled by unique DNA barcodes so that each cell can be distinguished after sequencing. Chromatin collected from single cells is pooled together for a conventional ChIP process. Carrier chromatin (i.e., chromatin extracted from other species) is used to minimize non-specific adsorption. They collected 7 × 10^6^ useful reads (700 000 unique reads) from 320 × 10^6^ reads in total (with a lot of reads consumed by carriers). On average, ∼1000 unique reads are obtained for each single cell. This represents the first successful microfluidic ChIP method for probing single cells.

#### 2. MeDIP and methyl-binding domain (MBD)

Affinity-based approaches, including Methylated DNA Immunoprecipitation sequencing (MeDIP-seq)^150,151^ and Methylated DNA Binding Domain sequencing (MBD-seq),^152^ are also used for probing DNA methylation. These technologies typically require substantial starting materials (1–300 ng).

Microfluidic devices can also be used to enrich methylated DNA by immunoprecipitation. Methyl-binding domain (MBD) protein^153,154^ or 5-methylcytidine (5-mC) antibody^127^ that specifically targets methylated DNA is immobilized on magnetic beads or the surface of the microfluidic chamber. When the DNA mixture contacts MBD protein or 5-mC antibody, methylated DNA is captured and enriched. These methods have been demonstrated for examining the methylation status of specific loci^127,155^ but have not been applied to genome-wide analysis.

#### 3. Transcription factor binding affinity

Transcription factors (TFs) are proteins that bind to specific DNA sequences so that they control the transcription of DNA. The most common way to profile transcription factor binding sites is by ChIP-seq. The TFs that bind to DNA are fixed to the genome by crosslinking. The chromatin is then fragmented by sonication and the TF/DNA complex is specifically selected by an antibody. ChIP-seq for TFs often requires more starting material than that for histone modification, due to less binding sites in the genome and lower efficiency for establishing TF/DNA connection by crosslinking. ChIP-seq for TFs has not been achieved on a microfluidic platform.

Maerkl *et al*. developed an alternative to systematically study the binding affinity of TFs, especially low-affinity interactions.^156–161^ The device contains 2400 units and each unit is controlled by three micromechanical valves and a “button” membrane. The button membrane is used for surface derivation and control molecular interaction. The chip surface is locally derived with antibody to capture target DNA and TFs. It provides a way for large-scale quantitative protein-DNA interaction measurement, which can be used to verify and predict the *in vivo* function of TFs.

Chen *et al*. developed a microfluidic device for SELEX Affinity Landscape MAPping (SELMAP) of TF binding.^162^ The device comprises 16 individually addressed reaction channels, each of which consists of 64 reaction chambers. TFs are immobilized on the surface of the microfluidic device by NeutrAvidin-biotin interaction. The target DNA oligos are captured by TFs and unbound oligos are digested by DNase. After DNase treatment, the remaining DNA is collected for PCR amplification and sequencing. The device allows measuring 16 proteins in parallel.

### C. Digestion-based approaches

The way that DNA is packaged into chromatin is critical for gene regulations. Several methods have been developed for analyzing chromatin conformation, accessibility, and nucleosome positioning.^163^ To evaluate the chromatin accessibility, specific enzymes (MNase, DNase and transposase) are used to digest chromatin. Combined with NGS, these methods are used for profiling genome-wide chromatin conformations. These methods include chromosome conformation capture (Hi-C),^164^ assay for transposase-accessible chromatin (ATAC-seq),^165^ DNase-seq,^166^ MNase-seq,^167^ and formaldehyde-assisted isolation of regulatory elements with sequencing (FAIRE-seq).^168^ Hi-C is used to identify the 3D conformation of chromatin. MNase-seq identifies the nucleosome positioning by digesting chromatin with MNase. In DNase-seq, chromatin is digested by DNase I. In ATAC-seq, DNA is fragmented and tagged by Tn5 transposase simultaneously.

These methods require hundreds of millions of cells as the starting material when the conventional versions were initially developed. In recent years, the sensitivities of these assays have been improved to the single cell level, including single cell Hi-C,^169^ single cell ATAC-seq,^130^ single cell DNase-seq,^170^ and single-cell MNase-seq.^171^ Single cell ATAC-seq was developed on a microfluidic platform. All these methods may potentially be implemented on microfluidic chips in order to improve the throughput and integration.

**FIG. 10.**
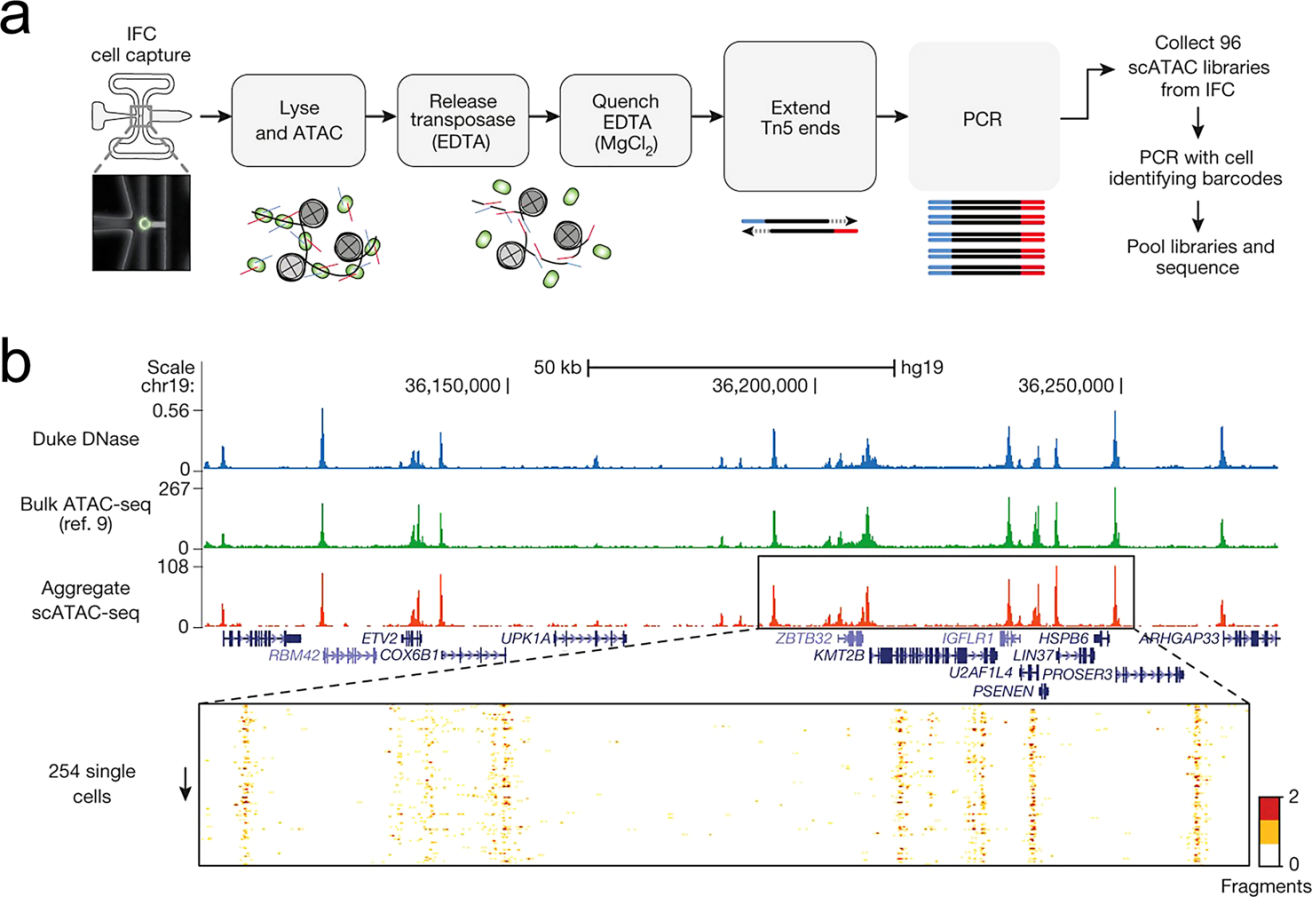
Single-cell ATAC-seq. (a) The workflow of scATAC-seq to measure single cell accessibility on a C1 microfluidic device (Fluidigm). (b) Aggregated single-cell accessibility profiles are similar to profiles of DNase-seq and ATAC-seq in GM12878 cells. Reprinted with permission from Buenrostro *et al*., Nature **523**(7561), 486–490 (2015). Copyright 2015 Macmillan Publishers Ltd.

Single cell ATAC-seq^130^ was operated on the C1 integrated fluidics circuit (IFC) by Fluidigm (Fig. 10). Individual cells are captured using “butterfly” single cell trapping structures. The cells are washed and stained for viability analysis. The cell membrane is then permeabilized by surfactant NP-40, and chromatin is treated with Tn5 transposase. Open chromatin is digested and tagged by Tn5 transposase, while closed chromatin remains intact. After transposition, the Tn5-DNA complexes are dissociated by adding EDTA. By performing 8 cycles of PCR, sequencing adapters are added onto the transposed DNA. Additional PCR cycles are used to amplify libraries in a 96-well plate (Fig. 10(a)). This method not only opened the door to chromatin accessibility analysis on a microfluidic chip, but also demonstrated an automated, parallel library preparation protocol starting with a limited amount of DNA. By Aggregating ATAC-seq signals from 254 single cells, the authors generated similar profiles to DNA-seq and bulk ATAC-seq. (Fig. 10(b))

## VII. LIBRARY CONSTRUCTION FOR NGS

The preparation of properly constructed sequencing libraries from DNA or RNA is still a time-consuming and tedious process. Most library preparation procedures are still manual and not suitable for high-throughput sample preparation, on top of the high cost. Microfluidics allows running parallel reactions/processes simultaneously and this makes it possible to streamline the entire library preparation. The ability to work with a small reaction volume (*μ*l to pl) may potentially improve the reproducibility. A typical library preparation procedure involves nucleic acid extraction, fragmentation, adapter ligation, amplification, and library quantification. Extracting high-quality DNA^2,172–175^ or RNA ^92,176–179^ from various species has been extensively covered in previous review articles.^9,180–185^

### A. DNA fragmentation

Depending on the process, the sizes of DNA templates are usually at least a few thousand base pair (bp) long, while NGS requires libraries with 200–600 bp length in order to bind to the sequencing flow cell. The DNA template needs to be fragmented before it is used for library construction. The most common methods are sonication and enzymatic fragmentation.

#### 1. Sonication

The conventional way to fragment DNA is using sonication. The ultrasonicator employs focused bursts of ultrasonic energy to a specific focal zone where numerous cavitation bubbles are generated. When each burst ends, these small bubbles collapse, create high velocity jets of solute, and break DNA into small fragments.

Tseng *et al*. described a DNA/chromatin shearing device within a microfluidic chip.^186^ The acoustic field is generated by attaching a piezoelectric Langevin-type composite transducer to a microfluidic chip. The fragment size can be controlled over a range from 180 to 4000 bp by adjusting voltages and pulse duration. Our group extended the work by adding crescent shaped structures in the microfluidic chamber, which improved generation of cavitation.^127^ We also integrated DNA/chromatin shearing (starting from fixed cells) with ChIP and MeDIP analysis (by qPCR). Such integration allowed highly sensitive assays with ∼100 cells for ChIP-qPCR and 500 pg DNA for MeDIP-qPCR.

#### 2. Enzymatic fragmentation

Fragmentase (NEBNext, New England Biolabs) and DNase I^187^ can also be used for DNA fragmentation. NEBNext dsDNA Fragmentase is the more popular choice. It contains a mix of two enzymes and generates DNA fragments of 100–800 bp in length by adjusting incubation time. The enzyme can be inactivated by heat at 65 °C for 15 min. Since there no additional equipment is needed, enzymatic fragmentation can be easily scaled up for high throughput library preparation. It has been employed in several applications including RNA-seq, DNA-seq, and haplotyping.^64,188^ It also showed the highest consistency among enzymatic fragmentation, sonication, and nebulization.^189^ Even though Fragmentase has not been used in a microfluidic chip, DNase has been implemented on chip^84^ for HIV genotyping. The device automatically conducted RNA purification, RT-PCR, nested PCR, DNase fragmentation, and hybridization to GeneChip oligonucleotide arrays.

### B. Ligation

In order to allow DNA fragments to attach to the flow cell, DNA needs to be ligated to adapters on both ends by ligase. When dealing with a limited amount of DNA, it is critical to ensure efficient ligation. Ligation among adapters (adapter dimers) reduces library quality.

Wook *et al*. designed the first microfluidic chip for DNA ligation, even though it was not specialized for NGS.^190^ DNA, vector, and enzyme are filled in three consecutive channels. These three solutions are pushed into a mixing ring and mixed by an actuating peristaltic pump. After incubating for 15 min, the DNA ligated to vector is eluted and ready for transformation. Similarly, Lin *et al*. used an electrowetting-on-dielectric (EWOD) microfluidic chip for DNA ligation with vectors.^191^ Reagents in their own reservoirs are separated into droplets, and the droplets are moved in the common microchannels and mixed. The solution is manipulated by electrical potential instead of an external pump or micromechanical valve. Ko *et al*. combined a micromixer with a microchannel reaction to reduce the complexity of the EWOD system.^192^

### C. Integrated library prep

Library preparation is a time-consuming and costly procedure. An automated sample preparation platform may help reduce assay time and reagent cost. A key step to perform library preparation is DNA purification. Enzyme, buffer, and small molecules used in each step need to be removed to avoid interference with subsequent steps. Kim *et al*. used a digital microfluidic (DMF) platform and AMPure XP magnetic beads to integrate multiple subsystem modules.^193^ AMPure XP beads bind to large DNA and exclude DNA smaller than a certain size, based on the buffer composition. DMF utilizes electrode arrays to transfer and merge liquid. It is programmed to exchange the buffer of the beads and wash the beads after binding. After this demonstration, they further adapted the entire tagmentation based Nextera library preparation protocol to their platform.^194^ *E. coli* genomic DNA (9 ng) is subjected to tagmentation (fragmentation and adding adapters), clean-up, PCR amplification, and size selection. The assay is finished in about 1 h with 5 min of hands-on time. Tan *et al*. designed an automated, multi-column chromatography (AMCC) chip to perform multiple purification on 16 independent samples.^195^ ChargeSwitch beads and AMPure beads are packed into a column. Both beads are capable of capturing/releasing DNA, depending on buffer composition. Peristaltic pumps were integrated to mix the samples with buffers and force samples to flow through the column for purification. Fagmented DNA (100 ng) was end-repaired, dA tailed, ligated with adapters, and size selected on chip. The assay was finished in about 4 h with 25 min hands-on time for 16 samples.

### D. Library quality control

Two major aspects for evaluating the quality of sequencing library are fragment size and library concentration.

#### 1. Library quantification

Depending on the sequencer and sequencing facility, libraries with 2–10 nM concentration are usually required for sample submission. Reliable quantification of library concentration will help obtain optimal amounts of reads during sequencing. A spectrometer (Nanodrop), a Fluorometer (Qubit), and quantitative PCR (KAPA library quantification system) have all been used for library quantification. Only DNA that is successfully ligated on both ends can be detected by quantitative PCR. Quantitative PCR provides better accuracy over the fluorometer and spectrometer. Digital PCR, a variation of quantitative PCR, calculates the absolute number of copies of DNA.^196^ The digital PCR has been conducted in either a micro-chamber^161,197^ or micro-droplet.^198^ It was demonstrated by White *et al*. that the digital PCR shows lower variation and higher sensitivity compared to real-time PCR based assays or spectrometer based assays.^196,199^ Digital PCR for measuring the DNA copy number has been intensively reviewed previously;^200–202^ thus it will not be further discussed in this review.

#### 2. Library fragment size

To determine the effective library concentration and verify the quality of the library, it is necessary to check the library fragment size (∼200–600 bp). Gel electrophoresis was the common way to determine the fragment size. Because of the minimal sample consumption and short assay time, microchip-based instruments (Bioanalyzer and Tapestation) are becoming more popular. Thaitrong *et al*. developed an automated platform for NGS quality control to improve upon these commercially available instruments.^203^ The system integrates a droplet-based digital microfluidic system, capillary-based reagent delivery unit, and quantitative capillary electrophoresis module. It is capable of measuring DNA of 5–100 pg/*μ*l and requires much less sample than Bioanalyzer.

## VIII. SUMMARY AND OUTLOOK

The use of microfluidics for NGS-related applications is still in its infancy. Significant efforts are still needed to develop mature platforms that complement individual assays and processes. Effective microfluidic platforms will help interface sample enrichment and preparation with sequencing library preparation. These efforts will be critical for tests of scarce primary cell samples required by precision medicine applications. Genome-wide studies using biomedically relevant samples will allow probing links among genomics, transcriptomics, and epigenomics. We summarize previous works related to microfluidic approaches for genome-wide analysis in Table I.

**TABLE I.** Currently available microfluidic approach for genome wide analysis.

Field	Approach	Reference	Format	Comment
Genomics	Digital PCR	56	Micro-chamber	1176 × 12 parallel reactions
	MDA	48, 49	Micro-chamber	Single cell, 9 parallel reactions
		50, 51	C1	Single cell, 96 parallel reactions
		46, 66	Droplet	Single cell
		52	Micro-well	Single cell, ∼400 cells per run
		53	Gel	Single cell
	MALBAC	55	Micro-chamber	Single cell, 8 parallel reactions
	PicoPLEX	54	Droplet	Single cell, 95 parallel reactions
Transcriptomics	T7 linear amplification	91	Micro-chamber	20 pg to 10 ng purified RNA
	SMART-seq	112–114	C1	Single cell, 96 parallel reactions
	Drop-seq	103	Droplet	Single cell, ≥1000 cells each run
	Hi-SCL	101	Droplet	Single cell, 10 000 cells in 4.3 h
	inDrop	102	Droplet	Single cell, ≥ 3000 cells
	Single-cell RNA printing and sequencing	100	Micro-well	Single cell, 600 cells
	CytoSeq	99	Micro-well	Single cell, up to 10 000 cells each run
	Tang's protocol^94^	107	Micro-chamber	Single cell, 8 parallel reactions
Epigenomics	ChIP-seq	128	Micro-chamber	1000 cells
	MOWChIP-seq	129	Micro-chamber	100 cells
	Drop-ChIP	131	Droplet	Single cell, 100 cells each run
	Transcription factors Binding affinity	156–161	Micro-chamber	2400 parallel reactions
	ATAC-seq	130	C1	Single cell, 96 parallel reactions

Future studies will potentially focus on implementing more genome-wide assays on microfluidic platforms. We will likely see major developments in several areas. First, droplet microfluidics will likely see more applications to single cell studies. We expect to see significantly improved data quality with these droplet assays in terms of the size of cell population surveyed and the amount of genome-wide information obtained from each cell. Second, epigenomic analysis is currently underdeveloped compared to genomic and transcriptomic analyses. We will potentially see increased effort in this area. Third, automated library preparation platforms remain to be fundamentally important for all NGS-related applications. More devices will be designed with parallel operations to improve throughput and automation. Together with advances in NGS and bioinformatics, these microfluidic tools will enable fundamental studies into genomics, transcriptomics, epigenomics, and their connections. Furthermore, these new tools will facilitate testing of scarce samples from small lab animals and patients and generate critical insights into molecular biology involved in development and diseases in the context of precision medicine.
